# Combined predictive value of dose, MODS, and NLR for lethal risk of diquat poisoning: a retrospective cohort study

**DOI:** 10.3389/ftox.2026.1897012

**Published:** 2026-10-05

**Authors:** Jifeng Dai, Meiyi He, Shi Chen, Yunchao Chen, Xin Li, Huaiman Ou, Yufan Deng, Caiying Zhong, Wenfeng Zeng, Yuquan Chen, Yanmei Ruan, Zhi Wang, Zejin Ou

**Affiliations:** 1 School of Public Health, North Sichuan Medical College, Nanchong, China; 2 School of Public Health, Southern Medical University, Guangzhou, China; 3 Department of Intensive Care Unit, Guangzhou Twelfth People's Hospital, Guangzhou, China; 4 School of Preschool Education and Healthcare, Xiangzhong Preschool Education College, Shaoyang, China; 5 Department of Internal Medicine-Cardiovascular, Guangzhou Twelfth People's Hospital, Guangzhou, China; 6 Occupational Health Surveillance Center, Guangzhou Occupational Diseases Prevention and Treatment Center, Guangzhou, China; 7 Department of Occupational Diseases and Poisoning, Guangzhou Twelfth People's Hospital, Guangzhou, China; 8 Key Laboratory of Occupational Environment and Health, Guangzhou Twelfth People's Hospital, Guangzhou, China; 9 Institute of Occupational and Environmental Health, Guangzhou Medical University, Guangzhou, China

**Keywords:** diquat, mortality, multiple organ dysfunction syndrome, neutrophil-to-lymphocyte ratio, self-poisoning

## Abstract

**Objective:**

Accurate risk assessment for fatal diquat poisoning is critical to improving clinical outcomes. Therefore, this study aimed to evaluate the combined value of the multiple organ dysfunction syndrome (MODS) and neutrophil-to-lymphocyte ratio (NLR) in predicting in-hospital all-cause mortality from acute diquat poisoning.

**Methods:**

This work retrospectively enrolled diquat self-poisoning patients in Guangzhou 12th People’s Hospital between 2017 and 2023. Demographic, exposure, and 24-h laboratory data were extracted. A time-dependent Cox model evaluated MODS as a time-varying predictor. LASSO and logistic regression identified independent predictors and established the prediction equation; restricted cubic splines assessed dose-response. Bootstrap internal validation evaluated discrimination and calibration; however, variable selection was not repeated within each resample, and therefore the optimism-corrected performance metrics should be interpreted with caution. SHAP quantified feature contributions.

**Results:**

NLR was significantly elevated in non-survivors compared to survivors (median 31.37 vs. 11.19, *P* < 0.001). In the time-dependent Cox analysis, MODS remained independently associated with mortality (hazard ratio [HR] = 35.24, 95% CI: 13.57–91.50, *P* < 0.001). In the exploratory logistic regression, MODS (adjusted odds ratio [aOR] = 34.63, 95% CI: 7.47–245.65, P < 0.001), diquat dose (aOR = 1.34 per 10 mL, 95% CI: 1.22–1.63, *P* < 0.001), and NLR (aOR = 1.07, 95% CI: 1.02–1.13, *P* = 0.01) were independently associated with in-hospital all-cause mortality. The combined model achieved an AUC of 0.976 (Bootstrap 95% CI: 0.951–0.998), with an optimism-corrected AUC of 0.970, an apparent calibration intercept of 0, an apparent calibration slope of 1, and a Brier score of 0.0553 (95% CI: 0.0140–0.0775). SHAP values ranked feature importance as diquat dose (0.179) > MODS (0.145) > NLR (0.069). Sensitivity analyses confirmed the robustness of these findings.

**Conclusion:**

MODS and NLR were independently associated with in-hospital all-cause mortality in acute diquat poisoning. When applied after the first 24–72 h of admission, the combined model shows promise as a potential exploratory tool for risk stratification; however, external validation in larger multicenter cohorts is required before any consideration of bedside application.

## Introduction

Pesticide self-poisoning was a substantial global challenge, which caused an estimated 168,000 deaths annually during the period of 2010 and 2014, accounting for 19.7% of global suicides (Mew et al., 2017). Diquat is a fast-acting bipyridine herbicide widely utilized in agricultural production (Cai et al., 2021; Zhang et al., 2021). Acute diquat poisoning was lethal, with high mortality rates reportedly ranging from 16.7% to 60% (Yan et al., 2023; Zhang et al., 2024). Early risk identification and precise intervention were essential for improving clinical outcome and reducing the mortality rate.

Acute diquat poisoning is characterized by rapid onset and progression, with severe manifestations involving the kidneys, liver, and central nervous system, potentially culminating in life-threatening multi-organ dysfunction (Feng et al., 2018; Feng and Lu, 2021; Meng et al., 2022). A case reported rapid onset of acute kidney injury, hemodynamic shock, and rhabdomyolysis following ingestion of only 40 mL of diquat (Chen et al., 2023). MODS is the leading cause of mortality in patients with acute diquat poisoning (Zhou and Lu, 2022; Li and Zhou, 2025). The toxicological mechanism of diquat is extremely complex, mainly involving excessive oxidative stress, mitochondrial dysfunction, and inflammatory activation, leading to severe multi-organ injury (Guo et al., 2023; Han et al., 2025). The clinical manifestations and mechanisms of diquat poisoning were inadequately understood, which highlighted the development of effective risk management approaches (Qian et al., 2025).

The neutrophil-to-lymphocyte ratio (NLR) was identified as an important biomarker of systemic inflammation, which had been widely used to predict the clinical outcome of sepsis (Suppiah et al., 2013; Song et al., 2021; Tang Q et al., 2025). An elevated NLR signified a serious immunological imbalance, which was strongly correlated with adverse prognoses of diquat poisoning (Magalhães et al., 2018; Tang Q et al., 2025; Tang H et al., 2025; Xue et al., 2025). However, systematic prognostic value of NLR in acute diquat poisoning remains relatively limited, requiring further exploration of its clinical utility.

Therefore, this study aimed to systematically assess the clinical characteristics of diquat self-poisoning and to establish a predictive model for in-hospital all-cause mortality. The findings would inform early identification of high-risk patients and optimize clinical risk stratification, providing an evidence-based reference for individualized monitoring and intervention.

## Materials and methods

This single-center retrospective cohort study was conducted at Guangzhou 12th People’s Hospital, the regional center for chemical poisoning treatment in Guangzhou, China. The study protocol was approved by the Clinical Research Ethics Committee of the hospital (No. 2023125) and fully complied with the Declaration of Helsinki. Given the retrospective nature of the study, which involved only the extraction of previous medical records, the requirement for informed consent was waived.

### Data collection

Clinical data of patients with diquat self-poisoning were retrospectively collected between May 2017 and May 2023. Inclusion criteria were: (1) confirmed history of diquat self-poisoning; (2) diquat identified as the sole causative agent; (3) estimated ingested dose >5 mL; (4) receipt of standardized treatment upon admission. Exclusion criteria were: (1) accidental poisoning; (2) death or discharge against medical advice within 24 h of admission without complete baseline clinical data; (3) severe pre-existing comorbidities, including end-stage renal disease or malignancies (4) detectable co-ingested toxins (e.g., paraquat or glyphosate); in blood or urine, with the identification of ultra-high performance liquid chromatography quadrupole-time of flight mass spectrometry (UPLC-QTOF/MS); (5) missing key clinical variables (Figure 1). Two independent researchers blinded to the study hypotheses extracted data using standardized forms and cross-verified the entries. The extracted data comprised demographic and clinical characteristics (age, sex, time from poisoning to admission, estimated diquat ingestion dose, and length of hospital stay). The ingestion dose was reported by the patients or their family members; when the dose was described in grams or milligrams, it was converted to milliliters using the concentration of the commercial 20% diquat solution (200 g/L), and all doses were uniformly expressed in milliliters throughout the analysis.

**FIGURE 1:**
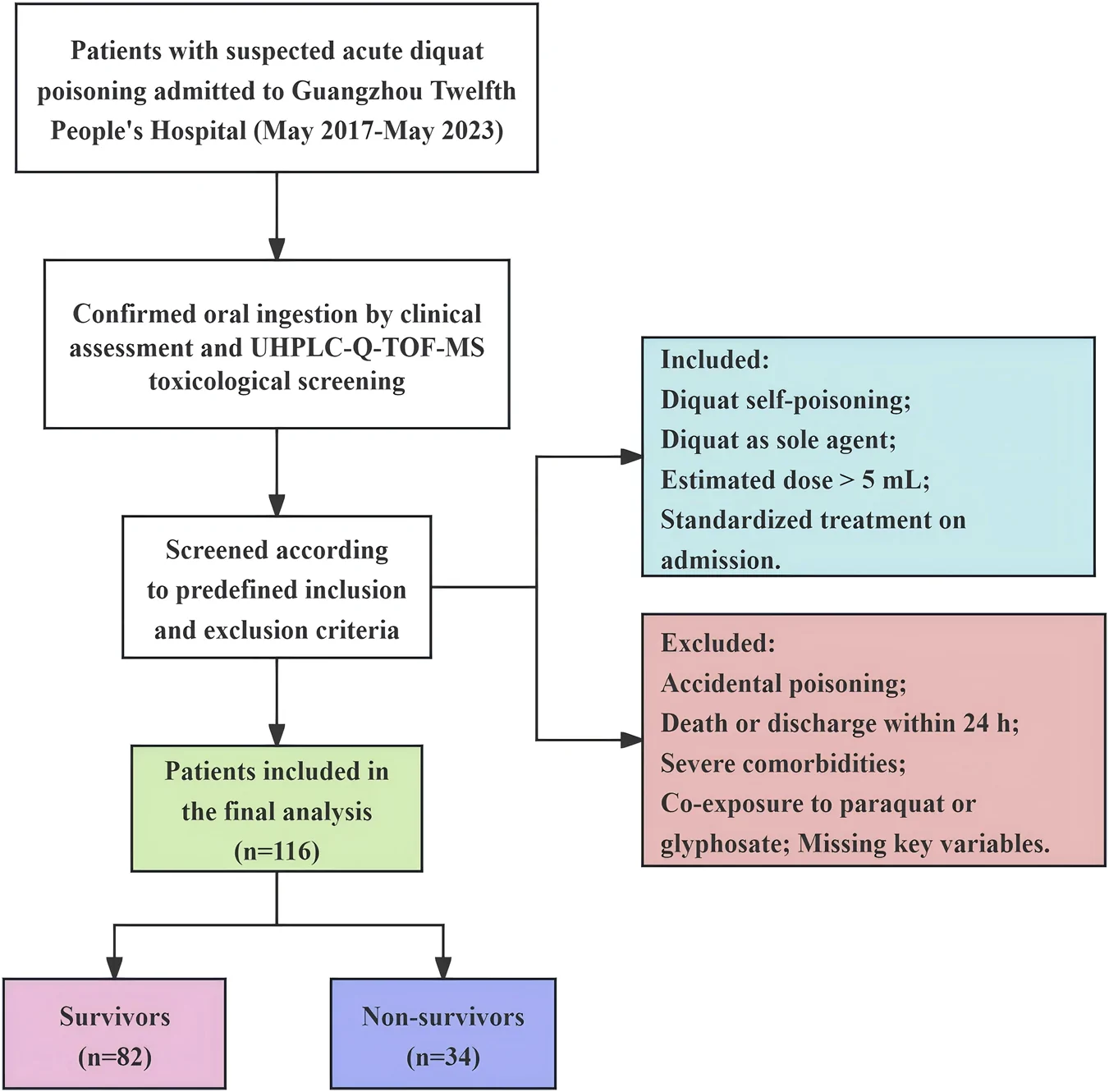
Flowchart of patient enrollment.

The primary outcome was in-hospital all-cause mortality, defined as death from any cause during the index hospitalization. Patients who were discharged alive were classified as survivors. All clinical complications, including MODS, were diagnosed within the first 72 h after ingestion and were thus considered early events occurring within the intended prediction window. MODS is a life-threatening clinical condition characterized by simultaneous dysfunction or failure of two or more organ systems, with the specific clinical code R65.301 of the International Classification of Diseases (10th Revision). The day of clinical confirmation was recorded and categorized into three ordered levels: the first 24 h, the second 24 h, and the third 24 h of admission. This diagnostic window was consistent with the 72-h acute phase (Zhang et al., 2017), supporting the reliability of MODS ascertainment within this temporal frame.

Composite inflammatory indices were calculated as previously described (Zhou et al., 2025): SII = NEU × PLT/LYM, NLR = NEU/LYM, PLR = PLT/LYM, LMR = LYM/MONO, NPR = NEU/PLT, SIRI = NEU × MONO/LYM, PAR = PLT/ALB, PIV = PLT × MONO × NEU/LYM, and HALP = HB × ALB × LYM/PLT.

### Analytical procedure and key parameters of UPLC-QTOF/MS

Analysis was performed using an Agilent 6545 Q-TOF/MS system with electrospray ionization (ESI) in positive ion mode. Chromatographic separation was carried out on a ZORBAX Hilic Plus column (3.0 × 100 mm, 1.8 μm) maintained at 40 °C. The mobile phase consisted of A (0.2% formic acid and 20 mM ammonium formate in water) and B (acetonitrile). The gradient elution program was as follows: 0–1 min, 2% A; 1–11 min, linearly increased to 75% A; 11–14 min, held at 75% A; 14.01–16 min, returned to 2% A. The flow rate was 0.4 mL/min and the injection volume was 3 μL. Mass spectrometric detection was performed with electrospray ionization (ESI) in positive ion mode, with a capillary voltage of 3500 V, a fragmentor voltage of 140 V, a drying gas temperature of 300 °C, a sheath gas temperature of 320 °C, and a mass scan range of m/z 20–1000. Real-time mass calibration was performed using reference ions at m/z 121.0509 and 922.0098. The acquisition was operated in All ions (MS/MS) mode, which alternated between low and elevated collision energies (0, 15, and 60 eV) to simultaneously acquire precursor and fragment ions.

### Statistical analysis

All statistical analyses were performed using R version 4.3.1. The normality of continuous variables was assessed using the Shapiro-Wilk test. Normally distributed variables are presented as mean ± SD and compared using the independent-samples t-test; skewed variables are presented as median (IQR) and compared using the Mann-Whitney U test. Categorical variables are presented as n (%) and compared using the χ^2^ test or Fisher’s exact test. A two-tailed *P* < 0.05 was considered statistically significant. No formal *a priori* sample size calculation was performed, as this study consecutively included all eligible patients during the study period. With 34 events, the logistic model was restricted to at most three covariates to maintain an events per variable ratio ≥10 (11.3), meeting the recommended minimum (Peduzzi et al., 1996).

For feature selection, LASSO regression (Ranstam J, 2018) was applied to 58 candidate variables after Z-score standardization, with λ determined by 10-fold cross-validation using λ.min and λ.1se criteria (glmnet package). Diquat dose and MODS were mandatorily included, and the third covariate was chosen from LASSO results. Under λ.1se, nine variables were identified; diquat dose, MODS, and NLR were retained under both λ criteria. Length of stay was excluded as a procedural outcome, and toxic hepatopathy/encephalopathy were omitted due to conceptual overlap with MODS. Given minor effect sizes of the remaining variables, NLR was selected. Multicollinearity was assessed by VIF (VIF >5 indicating significant collinearity). To address the potential concern of time-dependent prediction leakage, a time-dependent Cox regression analysis was first performed in which MODS was treated as a time-varying covariate. In this framework, patients were considered unexposed to MODS before the day of its diagnosis and exposed thereafter. This analysis served as the primary framework for evaluating the prognostic value of MODS, consistent with the principle that appropriate time-dependent modeling can effectively mitigate biases arising from data leakage (Rosenblatt et al., 2024). Logistic regression results were reported as OR with 95% CI. The final combined model equation was: logit(p) = −6.969 + 3.545 × MODS (Yes = 1, No = 0) + 0.030 × DQ (mL) + 0.064 × NLR, where p is the predicted probability of in-hospital all-cause mortality, calculated as p = 1/[1 + exp (−logit(p))] (Steyerberg, 2009). This model integrates exposure dose, terminal organ injury, and early inflammation into a synergistic predictive framework.

To improve interpretability, SHAP analysis (Van den Broeck G, 2022) was performed on the probability scale using fastshap (100 Monte Carlo simulations) and visualized via shapviz to generate feature importance, beeswarm, dependency, and waterfall plots. SHAP values represent the change in predicted probability attributable to each feature relative to the baseline risk. Four models were built (diquat dose alone, NLR alone, MODS alone, and combined). Discrimination was evaluated by AUC and compared with DeLong’s test; calibration was assessed by the Hosmer-Lemeshow test and calibration plot, and model performance was further quantified by AIC, BIC, and Brier score (DeLong et al., 1988). Internal validation used 1,000 bootstrap resamples (Efron B, 1994) to calculate the optimism-adjusted C-statistic and Brier score. The dose-response relationship between NLR and mortality was explored by restricted cubic spline (RCS) adjusted for MODS and diquat dose, with knots at the 10th, 50th, and 90th percentiles (3.60, 15.81, 44.28) and the 25th percentile (8.32) as reference; nonlinearity was tested by the likelihood ratio test (Durrleman and Simon, 1989). RCS and bootstrap analyses used the rms package, and ROC analyses used pROC. Sensitivity analyses (Frey et al., 2002) included log-transformation of NLR, exclusion of values above the 95th percentile, dichotomization at the median NLR, Firth penalized logistic regression, and calculation of the E-value to assess unmeasured confounding, all adjusted for MODS and diquat dose. Furthermore, Kaplan-Meier survival analysis was performed to compare survival distributions between patients stratified by the median NLR, with the log-rank test used to assess between-group differences.

## Results

### Clinical characteristics

A total of 116 patients with acute diquat poisoning were included in this study, comprising 34 (29.3%) non-survivors and 82 (70.7%) survivors. The clinical characteristics of the two groups were presented in Table 1. There were no significant differences in sex, age, or time from poisoning to first medical contact between the non-survivor and survivor groups (all *P* > 0.05). The non-survivor group ingested a significantly higher estimated dose of diquat than the survivor group (162.06 ± 66.50 mL vs. 52.15 ± 44.92 mL, *P* < 0.001) and had a significantly shorter length of hospital stay (median 1.00 days vs. 15.50 days, *P* < 0.001). Within 24 h of admission, both serum creatinine (median 229.00 vs. 126.50 μmol/L) and the NLR (median 31.37 vs. 11.19) were significantly higher in the non-survivor group compared with the survivor group (both *P* < 0.001).

**TABLE 1:** Baseline characteristics of patients with acute diquat poisoning stratified by Survivor status and MODS status.

Characteristics	Survivor N = 82^1^	Non-survivor N = 34^1^	*P*	Non-MODS N = 83^1^	MODS N = 33^1^	*P*
Gender						
Female	50 (61%)	16 (47%)	0.308	51 (61%)	15 (45%)	0.217
Male	32 (39%)	18 (53%)		32 (39%)	18 (55%)	​
Age (years)	24.15 (8.20)	23.65 (7.16)	0.760	24.37 (8.57)	23.06 (5.72)	0.619
Diquat ingestion dose (ml)	52.15 (44.92)	162.06 (66.50)	<0.001	58.68 (55.20)	148.18 (70.29)	<0.001
Time to local hospital after exposure (hours)	1.50 (1.00,4.00)	1.00 (1.00,3.00)	0.366	2.00 (1.00,5.00)	1.00 (1.00,2.00)	0.048
Hospitalization time (days)	15.50 (12.00,23.00)	1.00 (1.00,2.00)	<0.001	15.00 (11.00,23.00)	1.00 (1.00,2.00)	<0.001
Laboratory indicators						
HB	128.06 (21.87)	133.24 (24.52)	0.318	128.44 (21.27)	132.39 (26.13)	0.543
RBC	4.41 (0.77)	4.85 (0.87)	0.011	4.46 (0.74)	4.74 (0.99)	0.169
PLT	227.37 (103.25)	229.65 (110.13)	0.787	231.73 (101.93)	218.25 (113.01)	0.645
WBC	14.22 (6.98)	23.18 (7.73)	<0.001	14.69 (6.93)	22.23 (8.98)	<0.001
NEU	12.14 (6.79)	21.16 (6.74)	<0.001	12.59 (6.59)	20.24 (8.43)	<0.001
LYM	1.00 (0.62,1.63)	0.64 (0.42,1.08)	0.018	0.96 (0.60,1.63)	0.68 (0.46,1.28)	0.096
MONO	0.75 (0.48,1.14)	0.72 (0.42,1.38)	0.809	0.75 (0.45,1.14)	0.73 (0.49,1.38)	0.412
GT	17.00 (11.00,32.00)	18.50 (12.00,46.00)	0.484	19.30 (11.00,32.00)	17.00 (12.00,40.00)	0.830
ALT	19.25 (11.20,70.20)	182.20 (64.20,465.00)	<0.001	19.40 (11.20,61.90)	194.00 (70.40,374.80)	<0.001
AST	27.35 (18.30,45.20)	177.85 (45.40,372.60)	<0.001	27.50 (18.30,45.20)	171.50 (71.70,358.40)	<0.001
TB	11.95 (7.80,19.10)	9.90 (7.50,17.20)	0.385	11.20 (7.20,19.10)	11.40 (8.30,17.20)	0.988
DB	4.80 (3.40,6.30)	5.15 (3.90,6.50)	0.384	4.60 (3.40,6.30)	5.30 (4.00,6.50)	0.234
ALB	40.15 (8.96)	36.75 (13.82)	0.119	40.55 (9.66)	35.60 (12.24)	0.055
CK	100.00 (69.00,396.00)	1504.50 (406.00,3790.00)	<0.001	110.00 (69.70,522.00)	1,196.00 (199.00,3615.00)	<0.001
CKMB	23.05 (13.90,33.90)	104.35 (58.50,263.90)	<0.001	23.10 (13.90,36.00)	98.60 (43.10,346.30)	<0.001
LDH	256.50 (176.00,354.00)	1151.50 (560.00,1703.00)	<0.001	258.00 (176.00,361.00)	1,075.00 (556.00,1,703.00)	<0.001
HBDH	202.00 (141.00,293.00)	977.50 (438.00,1429.00)	<0.001	209.00 (141.00,305.00)	939.00 (293.00,1,429.00)	<0.001
Cr	126.50 (62.00,228.00)	229.00 (184.00,304.00)	<0.001	128.00 (62.00,304.00)	215.00 (164.00,260.00)	0.011
Urea	5.67 (3.97,11.67)	7.48 (6.39,10.33)	0.062	5.91 (3.97,12.09)	6.96 (6.05,8.34)	0.362
PT	11.65 (10.80,12.50)	12.95 (11.30,14.80)	0.012	11.90 (10.90,12.60)	11.80 (11.00,13.80)	0.206
TT	19.30 (17.80,22.20)	23.75 (18.80,150.00)	0.005	19.20 (17.60,21.60)	24.50 (19.00,150.00)	<0.001
APTT	25.80 (23.60,33.80)	30.20 (24.80,83.00)	0.027	25.70 (23.60,32.90)	31.50 (27.00,83.00)	0.005
FC	2.89 (2.26,3.61)	2.69 (1.96,3.54)	0.381	2.94 (2.27,3.72)	2.38 (2.11,3.23)	0.112
Dimer	0.88 (0.49,1.98)	2.50 (0.81,3.80)	0.007	0.87 (0.48,2.01)	1.98 (0.88,3.74)	0.008
K	3.72 (3.38,3.96)	4.05 (3.80,4.75)	<0.001	3.79 (3.38,4.09)	3.98 (3.68,4.44)	0.038
Na	137.35 (135.00,140.00)	139.50 (135.00,147.00)	0.078	138.00 (135.00,140.00)	139.00 (135.00,147.00)	0.129
CO2	20.50 (3.75)	14.58 (5.91)	<0.001	20.39 (3.85)	14.68 (5.99)	<0.001
AG	20.19 (17.64,23.25)	28.21 (23.69,30.66)	<0.001	20.55 (17.79,23.43)	27.42 (23.25,30.43)	<0.001
OP	277.57 (274.39,282.93)	283.94 (276.40,290.94)	0.025	277.65 (274.41,285.74)	281.00 (275.70,289.66)	0.160
NLR	11.19 (6.96,20.25)	31.37 (20.39,47.88)	<0.001	12.53 (7.21,21.79)	28.78 (14.17,40.20)	<0.001
NPR	0.05 (0.03,0.08)	0.09 (0.06,0.13)	<0.001	0.05 (0.03,0.08)	0.09 (0.07,0.13)	<0.001
LMR	1.52 (0.73,2.65)	0.77 (0.48,2.68)	0.067	1.60 (0.73,2.70)	0.77 (0.51,2.45)	0.053
PAR	5.61 (4.12,7.46)	5.72 (4.05,8.03)	0.308	5.59 (4.11,7.46)	5.84 (4.28,7.93)	0.355
PLR	209.85 (122.99,349.35)	324.79 (200.00,541.07)	0.017	242.71 (132.61,360.19)	246.67 (124.90,482.50)	0.386
PIV	1455.34 (632.57,3497.10)	3780.69 (1163.79,10504.40)	0.002	1,640.45 (664.02,3681.41)	3,594.46 (1074.96,10504.40)	0.030
SII	2263.35 (1229.67,4625.40)	5883.16 (3650.39,11608.70)	<0.001	2415.71 (1316.44,4892.80)	4948.20 (1987.49,11608.70)	0.011
SIRI	7.97 (2.84,17.46)	26.32 (5.45,46.24)	<0.001	7.99 (2.84,18.88)	23.27 (5.39,37.25)	0.002
HALP	25.19 (14.32,41.79)	13.97 (7.77,26.68)	0.005	25.19 (13.78,41.09)	15.53 (8.78,29.03)	0.065

1 n (%); Mean ± SD; Median (Q1,Q3);

2 Fisher’s exact test; Kruskal–Wallis rank sum test;

3 *P* values are descriptive and were not adjusted for multiple comparisons; they do not imply independent risk.

Among the numerous risk factors for mortality, MODS demonstrated the most prominent association with ingestion dose and systemic inflammation. To further elucidate the early predictive characteristics of MODS, all patients were stratified into two groups based on the occurrence of MODS (Table 1). The estimated diquat ingestion dose was significantly higher in the MODS group than in the non-MODS group (*P* < 0.001), accompanied by a longer length of hospital stay (*P* < 0.001). Furthermore, indicators of inflammation and organ damage within 24 h of admission, including white blood cell count, neutrophil count, liver enzymes, myocardial enzymes, and NLR, were also significantly elevated in the MODS group (all *P* < 0.05).

### LASSO regression screening and multivariable model construction

To identify potential predictors of all-cause mortality and to validate the combined predictive value of diquat dose, NLR, and MODS, LASSO regression was performed to screen 58 candidate variables. The cross-validation and coefficient shrinkage trajectories were illustrated in Figures 2A,B. Thirteen variables were retained under the λ.min criterion, which was reduced to nine variables under the λ.1se criterion. Diquat, NLR, and MODS maintained non-zero coefficients under both λ criteria. Notably, the standardized regression coefficient for NLR was 0.775, ranking highest among all retained inflammatory markers.

**FIGURE 2:**
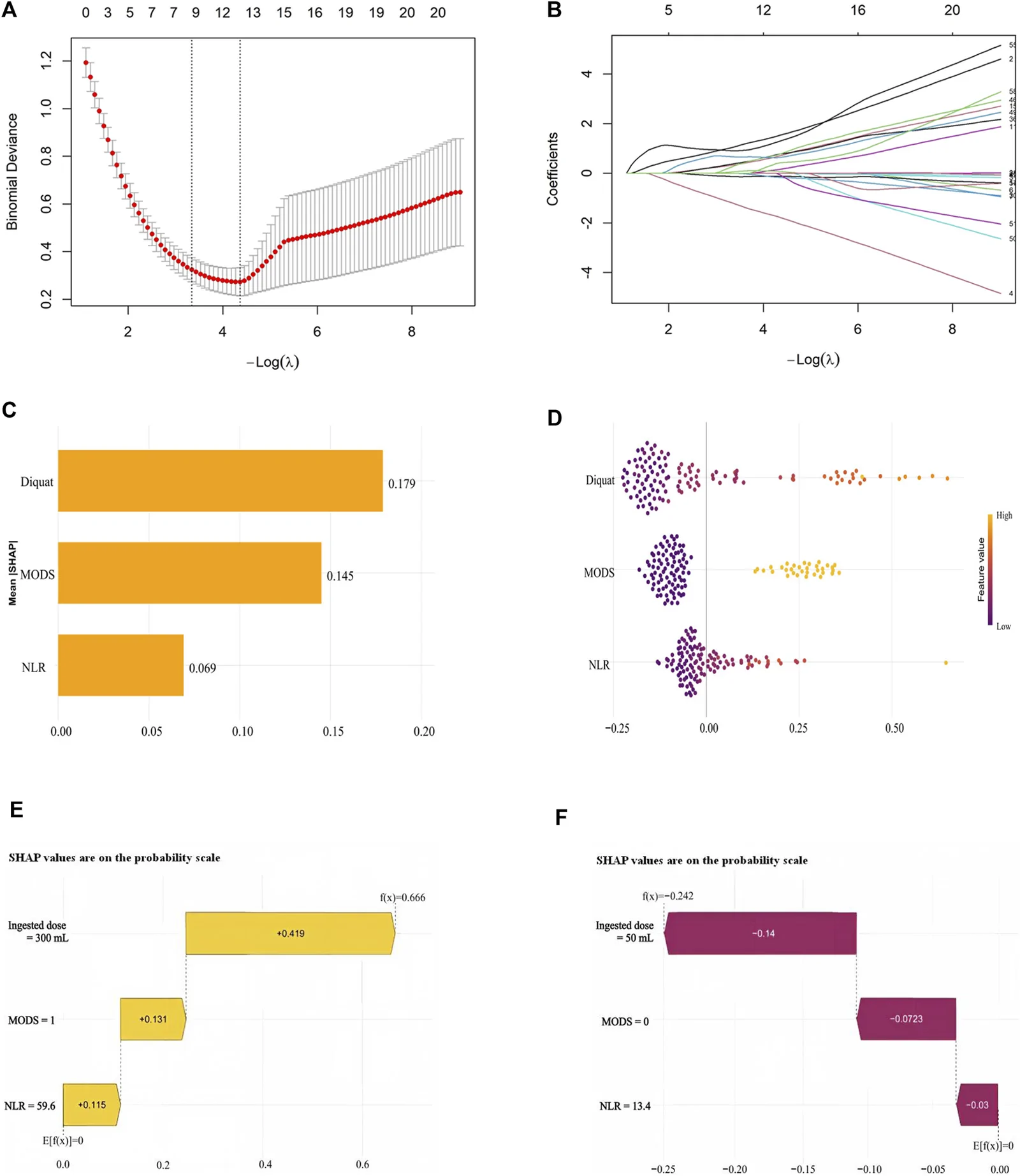
Variable selection and model interpretability for the combined predictive model. **(A)** 10-fold cross-validation curve for LASSO regression. **(B)** Coefficient path plot. **(C)** SHAP feature importance bar plot. **(D)** SHAP beeswarm plot. **(E)** SHAP waterfall plot for a survivor. **(F)** SHAP waterfall plot for a non-survivor. All SHAP values are expressed as changes in predicted probability.

Univariate logistic regression identified 28 variables significantly associated with the risk of mortality (Table 2). Among clinical complications, the association between MODS and mortality risk was the most striking (OR = 51.43). Toxic encephalopathy, acute respiratory distress syndrome, shock, and coma also significantly increased the risk of mortality (all *P* < 0.05; Table 2). Regarding laboratory indicators, both NLR and Diquat were significantly and positively associated with mortality risk. Additionally, NLR was significantly associated with the risk of developing MODS (OR = 1.047, *P* < 0.001), demonstrating the strongest correlation among all composite inflammatory biomarkers (Table 3).

**TABLE 2:** Univariable and multivariable associations between clinical complications and all-cause mortality.

Characteristics	Diagnosis	Survivor group N (%)	Non-survivor group N (%)	OR (univariate)	*P*	OR (multivariable)	*P*
Total (n = 116)	​	82 (70.7%)	34 (29.3%)	​	​	​	​
Toxic nephropathy	0	36 (43.9%)	9 (26.5%)	​	​	​	​
	1	46 (39.7%)	25 (73.5%)	2.42 (1.04–6.03)	0.047	0.46 (0.07–3.05)	0.425
Acute kidney injury	0	72 (87.8%)	25 (73.5%)	​	​	​	​
	1	10 (12.2%)	9 (26.5%)	2.74 (0.98–7.56)	0.050	0.15 (0.02–1.31)	0.09
Toxic lung injury	0	59 (72.0%)	24 (70.6%)	​	​	​	​
	1	23 (28.0%)	10 (29.4%)	1.14 (0.46–2.71)	0.768	​	​
Toxic myocardial injury	0	74 (90.2%)	17 (50%)	​	​	​	​
	1	8 (9.8%)	17 (50%)	9.75 (3.73–27.54)	<0.001	0.40 (0.03–5.35)	0.485
Toxic encephalopathy	0	64 (78.0%)	7 (20.6%)	​	​	​	​
	1	18 (22.0%)	27 (79.4%)	14.57 (5.73–41.52)	<0.001	12.14 (1.92–76.92)	0.008
Acute pancreatitis	0	80 (97.6%)	29 (85.3%)	​	​	​	​
	1	2 (2.4%)	5 (14.7%)	7.24 (1.47–52.51)	0.022	0.14 (0.01–3.34)	0.222
Toxic pancreatic injury	0	79 (96.3%)	28 (82.4%)	​	​	​	​
	1	3 (3.7%)	6 (17.6%)	5.93 (1.46–29.58)	0.016	0.19 (0.01–3.01)	0.237
Acute respiratory distress syndrome	0	80 (97.6%)	22 (64.7%)	​	​	​	​
	1	2 (2.4%)	12 (35.3%)	22.91 (5.72–154.41)	<0.001	5.20 (0.60–45.44)	0.136
Rhabdomyolysis	0	79 (96.3%)	32 (94.1%)	​	​	​	​
	1	3 (3.7%)	2 (5.9%)	1.73 (0.22–10.90)	0.559	​	​
Multiple organ dysfunction syndrome	0	76 (92.7%)	7 (20.6%)	​	​	​	​
	1	6 (7.3%)	27 (79.4%)	51.43 (17.10–182.93)	<0.001	145.66 (14.97–1417.69)	<0.001
Shock	0	78 (95.1%)	24 (70.6%)	​	​	​	​
	1	4 (4.9%)	10 (29.4%)	8.54 (2.61–33.44)	<0.001	1.04 (0.16–6.82)	0.964
Brain Hernia	0	80 (97.6%)	29 (85.3%)	​	​	​	​
	1	2 (2.4%)	5 (14.7%)	7.24 (1.47–52.51)	0.022	0.06 (0.00–0.86)	0.039
Coma	0	80 (97.6%)	26 (76.5%)	​	​	​	​
	1	2 (2.4%)	8 (23.5%)	12.92 (3.01–89.29)	0.002	5.04 (0.54–46.63)	0.154

Data were presented as OR (univariable) or aOR (multivariable) with 95% CI., The multivariable model included all clinical complications listed in the table and mutually adjusted for one another. OR, odds ratio; aOR, adjusted odds ratio; CI, confidence interval.

**TABLE 3:** Univariable associations of laboratory parameters with all-cause mortality and MODS.

Laboratory indicators	Survivor or non-survivor		MODS or No-MODS	
	OR (Univariate)	*P*	OR (Univariate)	*P*
HB	1.010 (0.993–1.029)	0.260	1.008 (0.990–1.027)	0.393
RBC	2.056 (1.215–3.692)	0.010	1.538 (0.931–2.637)	0.102
PLT	1.002 (0.996–1.004)	0.914	0.999 (0.995–1.003)	0.528
WBC	1.175 (1.103–1.266)	<0.001	1.132 (1.070–1.207)	<0.001
NEU	1.202 (1.122–1.305)	<0.001	1.151 (1.083–1.235)	<0.001
LYM	0.736 (0.399–1.259)	0.292	0.809 (0.445–1.373)	0.457
MONO	1.387 (0.791–2.482)	0.247	1.25 (0.701–2.207)	0.429
GT	1.006 (0.997–1.016)	0.190	1.001 (0.991–1.011)	0.775
ALT	1.004 (1.002–1.007)	0.001	1.004 (1.002–1.007)	0.002
AST	1.002 (1.001–1.005)	0.006	1.003 (1.001–1.005)	0.005
TB	1.001 (0.994–1.008)	0.679	1.002 (0.994–1.008)	0.588
DB	1.002 (0.994–1.011)	0.517	1.003 (0.994–1.011)	0.466
ALB	0.971 (0.933–1.007)	0.119	0.957 (0.918–0.994)	0.028
CK	1.003 (1.001–1.006)	0.006	1 (1.000–1.000)	0.008
LDH	1.001 (1.001–1.002)	<0.001	1.001 (1.001–1.002)	<0.001
HBDH	1.001 (1.001–1.002)	<0.001	1.001 (1.001–1.002)	<0.001
Cr	1.002 (1.001–1.004)	0.040	1.001 (0.999–1.004)	0.162
Urea	1.022 (0.971–1.074)	0.388	1.016 (0.963–1.067)	0.54
PT	1.008 (0.958–1.054)	0.707	1.046 (0.996–1.141)	0.177
TT	1.013 (1.006–1.021)	<0.001	1.015 (1.007–1.022)	<0.001
APTT	1.009 (1.001–1.017)	0.021	1.01 (1.002–1.017)	0.013
FC	1.004 (0.999-NA)	0.787	1.003 (1.000-NA)	0.728
Dimer	0.999 (0.944–1.042)	0.989	1.008 (0.959–1.051)	0.719
K	3.742 (1.954–8.640)	<0.001	2.059 (1.256–3.693)	0.007
Na	0.999(NA-1.003)	0.685	0.999(NA-1.003)	0.694
CO2	0.779 (0.699–0.856)	<0.001	0.791 (0.712–0.867)	<0.001
AG	1.310 (1.183–1.485)	<0.001	1.192 (1.104–1.309)	<0.001
OP	1.003 (0.995–1.017)	0.559	0.997 (0.990–1.006)	0.492
NLR	1.071 (1.040–1.107)	<0.001	1.047 (1.021–1.077)	<0.001
NPR	57805.22 (31.240–281419005.87)	0.008	69.512 (2.672–71674.635)	0.152
LMR	0.952 (0.808–1.063)	0.459	0.932 (0.773–1.050)	0.35
PAR	1.010 (0.994–1.033)	0.235	1.01 (0.994–1.032)	0.236
PLR	1.003 (1.001–1.005)	0.005	1.002 (1.000–1.004)	0.095
PIV	1.0001 (1.00004–1.00019)	0.005	1 (1.000–1.000)	0.007
SII	1.0002 (1.0001–1.0003)	<0.001	1 (1.000–1.000)	0.002
SIRI	1.038 (1.019–1.062)	<0.001	1.028 (1.011–1.048)	0.002
HALP	1.0005 (0.9999-NA)	0.502	1 (1.000-NA)	0.518

Data are presented as OR, with 95% CI., All laboratory parameters were measured within 24 h of admission. Variables are reported as mean ± SD, or median (IQR) depending on the normality of their distributions. Where an OR, is displayed as 1.000 with a 95% CI, of 1.000–1.000, this reflects rounding to three decimal places and does not indicate a precisely null effect. MODS, multiple organ dysfunction syndrome.

At the multivariable level, all clinical complications were first simultaneously entered into a single model for mutually adjusted analysis. Only MODS (adjusted OR [aOR] = 145.66, 95% CI: 14.97–1417.69, *P* < 0.001) and toxic encephalopathy (aOR = 12.14, 95% CI: 1.92–76.92, *P* = 0.008) retained statistical significance (Table 2), underscoring the central role of MODS in the terminal injury stage. Building on this finding, and guided by the dual-λ LASSO criteria and the EPV ≥10 rule, a parsimonious model incorporating diquat dose, MODS, and NLR was constructed. Multivariable logistic regression confirmed that all three variables were independent risk factors for all-cause mortality (Table 4). The odds of death increased by 34% per 10 mL increase in estimated ingested dose (aOR = 1.34, 95% CI: 1.22–1.63, *P* < 0.001); the presence of MODS was associated with a 34-fold increase in mortality risk (aOR = 34.63, 95% CI: 7.47–245.65, *P* < 0.001); and each one-unit increase in NLR was associated with a 7% increase in mortality risk (aOR = 1.07, 95% CI: 1.02–1.13, *P* = 0.01).

**TABLE 4:** Multivariable logistic regression of the combined Diquat + MODS + NLR model for mortality.

Characteristics	OR (95% CI)	*P*
Diquat ingestion dose (per 10 mL increase)	1.34 (1.22–1.63)	<0.001
Multiple organ dysfunction syndrome (yes vs. no)	34.63 (7.47–245.65)	<0.001
NLR (per 1 unit increase)	1.07 (1.02–1.13)	0.01

OR, odds ratio; CI, confidence interval. The model was adjusted for all variables listed in the table.

For clinical application, the final combined logistic regression equation is as follows:

$$\text{logit}\left(p\right)=‐6.969+3.545\times \text{MODS }\left(\text{Yes}=1,\text{No}=0\right)+0.030\times \text{DQ }\left(\text{mL}\right)+0.064\times \text{NLR}$$

where p is the predicted probability of in-hospital all-cause mortality. The predicted probability can be calculated as p = 1/[1 + exp (−logit(p))].

### SHAP-based interpretability analysis of the multivariable logistic regression model

SHAP analysis was performed on the probability scale to complement the odds ratios from the logistic regression model (Figures 2C–F). Feature importance ranking was consistent with the multivariable results: diquat dose contributed most (mean |SHAP| = 0.179), followed by MODS (0.145) and NLR (0.069) (Figure 2C). The beeswarm plot illustrated that high diquat dose and the presence of MODS shifted the predicted probability upward, with NLR showing a similar but weaker pattern (Figure 2D). Waterfall plots for representative patients decomposed individual predictions on the probability scale. For a non-survivor (diquat dose 300 mL, MODS positive, NLR 59.6), the predicted probability was 0.666, with diquat dose contributing +0.419, MODS contributing +0.131, and NLR contributing +0.115 relative to the baseline risk (Figure 2E). For a survivor (diquat dose 50 mL, MODS negative, NLR 13.4), the predicted probability was 0.000, with diquat dose contributing −0.140, MODS contributing −0.072, and NLR contributing −0.030 (Figure 2F). All SHAP values are expressed as changes in predicted probability.

### Time-dependent cox analysis of MODS

To formally address the potential concern of time-dependent prediction leakage, a time-dependent Cox regression was performed with MODS treated as a time-varying covariate. In this framework, patients were considered unexposed to MODS before the day of its diagnosis and exposed thereafter. MODS remained independently associated with mortality (HR = 35.24, 95% CI: 13.57–91.50, *P* < 0.001). Diquat dose retained a significant association (HR = 1.007 per 1 mL, 95% CI: 1.002–1.012, *P* = 0.011), and NLR showed a consistent positive trend (HR = 1.015, 95% CI: 0.996–1.033, *P* = 0.119). The baseline model (DQ + NLR) achieved a time-dependent AUC of 0.932 at 30 days, and Schoenfeld residual tests indicated no violation of the proportional hazards assumption (all *P* > 0.05) (Supplementary Table S1; Supplementary Figure S1). These effect estimates were closely concordant with those from the logistic regression model.

### Model performance evaluation and dose-response relationship

For the purpose of establishing a clinically applicable prediction equation, exploratory logistic regression models were also constructed. Four predictive models were built: three individual models based on diquat dose, NLR, and MODS alone, and one combined model. The receiver operating characteristic (ROC) curves are presented in Figure 3A. Among the single indicators, diquat dose exhibited the highest area under the curve (AUC) (0.907, 95% CI: 0.854–0.959), followed by MODS (0.862, 95% CI: 0.788–0.936) and NLR (0.806, 95% CI: 0.715–0.898). The combined model achieved an AUC of 0.976 (Bootstrap 95% CI: 0.951–0.998). DeLong’s test indicated that the discriminative ability of the combined model was significantly superior to each individual model (diquat dose: ΔAUC = 0.069, *P* = 0.005; NLR: ΔAUC = 0.169, P < 0.001; MODS: ΔAUC = 0.114, *P* < 0.001).

**FIGURE 3:**
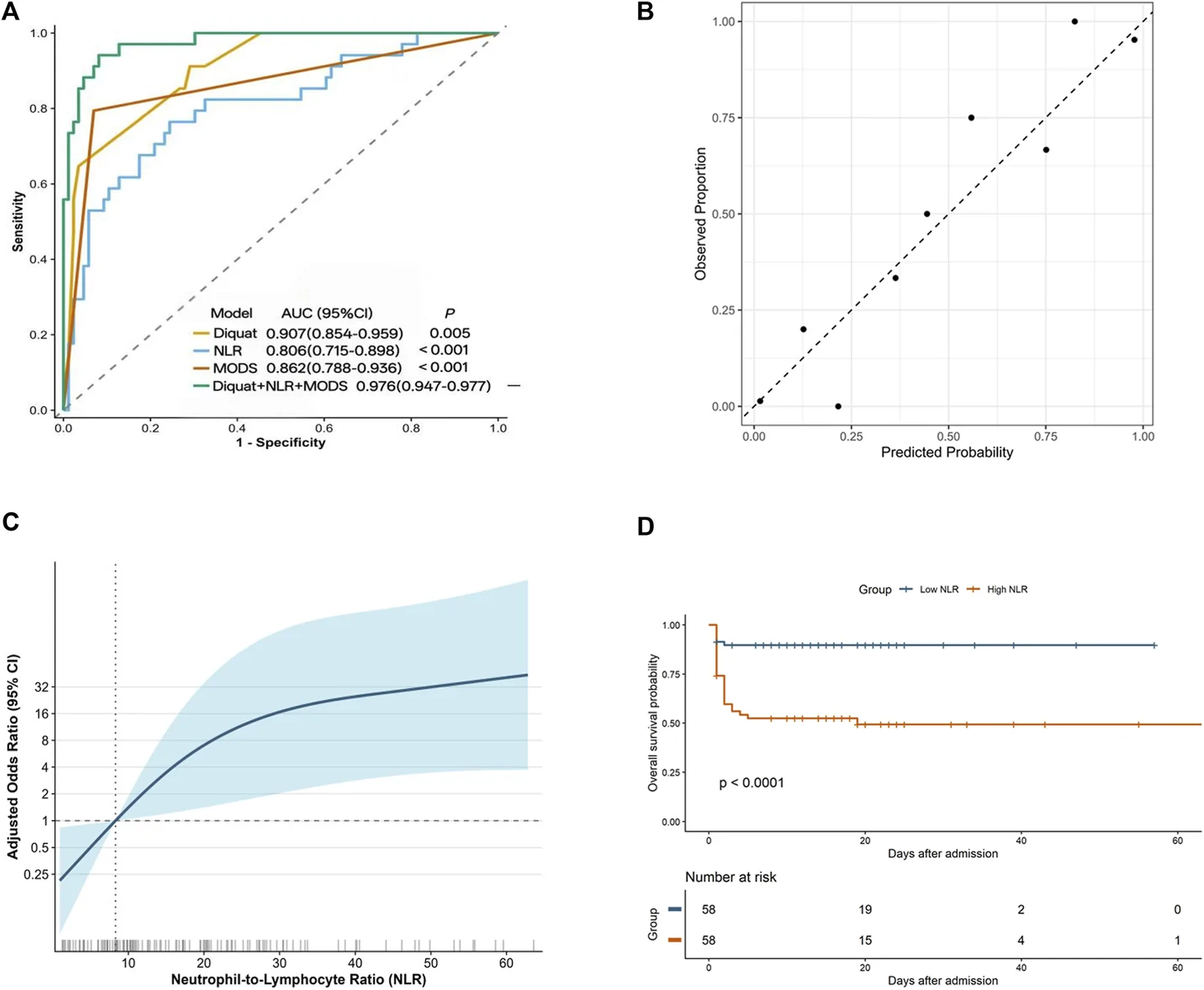
Predictive performance, calibration, dose-response relationship, and survival analysis. **(A)** ROC curves of the four predictive models. **(B)** Calibration plot of the combined model. **(C)** Adjusted dose-response relationship between NLR and mortality. **(D)** Kaplan-Meier survival curves stratified by the median NLR.

Calibration of the combined model was assessed using a calibration plot (Figure 3B), which demonstrated close agreement between predicted probabilities and observed outcomes. On apparent assessment, the calibration intercept was 0 and the calibration slope was 1, both at their ideal values, although these estimates were not adjusted for optimism via bootstrap resampling. The Brier score was 0.0553 (Bootstrap 95% CI: 0.0140–0.0775), further supporting satisfactory calibration. A restricted cubic spline (RCS) model, fitted with three knots at the 10th, 50th, and 90th percentiles, was utilized to evaluate the dose-response relationship between NLR and mortality risk, adjusting for MODS and diquat dose (Figure 3C). Using an NLR of 8.32 as the reference, the adjusted odds ratio (aOR) increased progressively with rising NLR, with a suggestion of attenuation in the upper range that did not reach statistical significance. At an NLR of approximately 60, the aOR reached 10.43 (95% CI: 1.16–93.52); the widened confidence intervals in the high-NLR segment (>40) were attributable to the limited sample size. The Wald test demonstrated a significant overall association between NLR and mortality (χ^2^ = 9.42, *P* = 0.009). The formal test for nonlinearity was not statistically significant (*P* = 0.095); therefore, a linear assumption was considered acceptable for the present data.

### Sensitivity analysis

The results of the sensitivity analyses were detailed in Table 5. In the primary model, the aOR for NLR was 1.07 (95% CI: 1.02–1.13). Following log-transformation (aOR = 7.70, 95% CI: 2.44–34.27), exclusion of extreme values above the 95th percentile (aOR = 1.13, 95% CI: 1.05–1.24), and dichotomization at the median (aOR = 12.72, 95% CI: 2.39–103.89), the positive association between NLR and mortality risk remained directionally consistent and statistically significant across all scenarios (all *P* < 0.05). Given the low incidence of MODS in survivors (7%), which may lead to separation and unstable estimates in standard logistic regression, we additionally performed Firth penalized logistic regression as a sensitivity analysis. The results confirmed that MODS (aOR = 24.87, 95% CI: 6.11–140.78), DQ (aOR = 1.027, 95% CI: 1.015–1.043), and NLR (aOR = 1.056, 95% CI: 1.018–1.114) remained independent risk factors (all *P* < 0.01), with the MODS estimate being more stable and its confidence interval substantially narrower than that from conventional logistic regression. The E-value analysis based on the binary NLR contrast (high vs. low, median cutoff) yielded a point estimate of 24.9 and a lower confidence limit E-value of 4.2, indicating that an unmeasured confounder would need to be associated with both NLR and mortality by a risk ratio of at least 24.9-fold to fully explain the observed 12.72-fold mortality risk, and by at least 4.2-fold to shift the confidence interval to include the null. This suggests reasonable robustness to unmeasured confounding for this clinically meaningful contrast. Overall, while the prognostic association of NLR remained robust across different modeling specifications, these findings warrant cautious interpretation in the context of residual confounding and the observational study design. Kaplan-Meier analysis stratified by the median NLR (15.81) demonstrated that patients in the high NLR group had significantly shorter survival compared with those in the low NLR group (log-rank *P* < 0.001; Figure 3D). The survival curve for the high NLR group declined steeply within the first 10 days after admission, with overall survival probability dropping to approximately 55%, whereas the low NLR group maintained an overall survival probability above 90% throughout the observation period.

**TABLE 5:** Sensitivity analyses of the association between NLR and all-cause mortality.

Analysis	OR (95% CI)	*P* value
Primary (NLR linear)	1.07 (1.02–1.13)	0.010
Log (NLR) transformation	7.70 (2.44–34.27)	0.002
Trimmed (NLR≤95th percentile)	1.13 (1.05–1.24)	0.003
Binary (median cutoff, high vs. low)	12.72 (2.39–103.89)	0.007
Firth penalized logistic regression		
MODS	24.87 (6.11–140.78)	<0.001
DQ (per 10 mL)	1.34 (1.22–1.63)	<0.001
NLR (per 1 unit)	1.056 (1.018–1.114)	0.003
E-value (binary NLR, high vs. low)	Point estimate: 24.9; CI lower limit: 4.2	-

All models were adjusted for diquat dose and Multiple Organ Dysfunction Syndrome (MODS), unless otherwise specified. OR, odds ratio; CI, confidence interval. The E-value for the binary NLR, contrast was calculated based on the aOR, of 12.72 and its 95% CI, lower limit of 2.39.

## Discussion

This study systematically evaluated the clinical characteristics of diquat self-poisoning. Through a time-dependent Cox analysis, we confirmed that MODS remains independently associated with mortality after accounting for its time-varying nature. An exploratory logistic regression model was then used to establish a clinical prediction equation. Because the logistic equation directly yields a probability formula, it can be readily applied at the bedside, whereas the time-dependent Cox model provides effect estimates without a simple prediction formula. The logistic model demonstrated the combined prognostic value of MODS and NLR. Low-dose ingestion typically causes mild gastrointestinal symptoms with good prognosis, whereas moderate-to-severe poisoning often progresses rapidly to MODS and high mortality (Zhang et al., 2020). To ensure clinical homogeneity and focus on patients at definitive risk, we restricted inclusion to an estimated ingested dose >5 mL, avoiding dilution of model performance by inherently favorable cases.

MODS had been demonstrated as the critical factor of mortality in acute pesticide poisoning. Its reported incidence and associated mortality vary across studies due to differences in patient severity and sample size. For example, Huang K et al. (2020) reported a MODS incidence of 72.06% in a diquat poisoning cohort, with mortality rising to 95.92% in patients with MODS versus 47.37% in patients without MODS. In the present study, MODS incidence was greatly higher among non-survivors than survivors, aligning closely with those previous studies (Zhang et al., 2026). A hallmark distinguishing diquat from paraquat poisoning is this sequential injury trajectory, which initially affects the kidneys and liver before spreading systemically (Ren et al., 2026). MODS is highly associated with the mortality risk, thus further identifying its modifiable drivers (e.g., excessive inflammation) becomes a key priority for risk management.

NLR integrated innate and adaptive immunity; its elevation reflects a severe imbalance between pro-inflammatory activity and immunosuppression, making this convenient marker well-suited to capture early warning signals preceding MODS from an immunological perspective (He et al., 2023). Diquat had strong capacity to induce cellular death and organ injury by oxidative stress and mitochondrial damage, leading to the release of damage-associated molecular patterns (DAMPs) that recruit and activate neutrophils (Choi et al., 2018; Konecna et al., 2020). These activated neutrophils subsequently release excessive ROS agents, proteases, and NETs, thereby amplifying systemic inflammation (Papayannopoulos, 2018). At the same time, stress hormones (cortisol, catecholamines) induce lymphocyte apoptosis (Migita et al., 1997), and pro-apoptotic signaling further depletes lymphocytes (Hotchkiss et al., 2013). Thus, an elevated NLR encapsulates a state of concurrent pro-inflammatory overactivation and immunosuppression, which is closely associated with rapid MODS progression, providing strong biological plausibility for NLR as a prognostic biomarker (Akilli et al., 2014). Despite its well-validated prognostic role in sepsis and acute pancreatitis (Huang Z et al., 2020; Lu et al., 2023), NLR remains understudied in diquat poisoning (Liu et al., 2023). Prior studies noted higher NLR in non-survivors; nevertheless, neither study adjusted for key confounders, assessed continuous dose-response relationships, nor underwent rigorous model validation (Tang H et al., 2025; Wang et al., 2025). Our study advances these findings through LASSO screening, time-dependent Cox regression to rigorously evaluate MODS while addressing prediction leakage, multivariable logistic regression for prediction equation derivation, RCS for nonlinear dose-response exploration, SHAP for interpretability, and internal bootstrap validation with multidimensional sensitivity analyses. The combined model demonstrated excellent discrimination, with the AUC of the diquat-only model improved significantly upon the integration of MODS and NLR, indicating that NLR provided independent incremental value to predictive frameworks. Moreover, NLR was robustly retained under both λ.min and λ.1se in the LASSO analysis framework, whereas other composite inflammatory indices such as SII, PLR, and SIRI were shrunk to zero. Although previous studies had suggested prognostic roles for SII/SIRI in pesticide poisoning (Xie et al., 2024; Şahin et al., 2025), their independent contribution was outweighed by NLR in this cohort, reaffirming the unique predictive power of NLR. The findings provided preliminary evidence for NLR in early risk stratification of diquat poisoning. As a routine complete blood count derivative, NLR is cost-effective and rapidly accessible, enabling its clinical application to be extended to emergency and intensive care (Li et al., 2014). Sensitivity analyses revealed that patients with NLR above the median of 15.81 had a 12.72-fold higher mortality risk, suggesting NLR >16 as a pragmatic early warning threshold. This finding is in line with prior data on diquat poisoning, where non-survivors exhibited comparably elevated NLR levels: a study of 117 patients reported a median NLR of 15.6 in the non-survivor group, with a 1.101-fold increased mortality risk per unit rise in NLR (OR = 1.101) (Tang Q et al., 2025), and another cohort of 145 patients documented a median NLR of 18 among non-survivors (Wang et al., 2025). These consistent observations reinforce the reliability of NLR >16 as an early warning indicator of poor outcome. Further SHAP waterfall plots enable individualized risk attribution, allowing clinicians to intuitively assess each prognostic factor’s contribution. Previous studies had established a mortality risk prediction model for diquat poisoning (Lv and Du, 2024). In contrast, this study systematically integrated MODS, diquat dose, and NLR into a combined model. When applied after the first 24–72 h of admission, once MODS status has been ascertained, the model shows promise as a potential bedside risk-assessment tool, pending external validation. The Kaplan-Meier curves further reinforced this point: patients with high NLR exhibited a rapid decline in survival within the first 10 days, whereas those with low NLR had a relatively favorable short-term prognosis, underscoring the importance of early NLR-based risk stratification to identify patients who may benefit from intensified monitoring and intervention shortly after admission.

This study has several limitations. First, the single-center retrospective design and the relatively small number of events (34) limit the generalizability of the findings. Given the rarity of acute diquat poisoning, this cohort represents one of the larger single-center samples reported to date. Although the events per variable ratio met the recommended minimum, the model should be regarded as exploratory rather than definitive, and external validation in larger multicenter cohorts is essential before any clinical application can be considered. Second, four patients who died or were discharged within 24 h of admission were excluded because their baseline data were incomplete. This exclusion may introduce survival bias by omitting hyper-acute cases, and the model may therefore underestimate the risk of very early death. The intended use of the model is risk stratification after the first 24–72 h, once baseline data are complete and MODS status can be determined. Third, the estimated ingested dose relied on patient or family recall and is subject to unavoidable measurement error, particularly when consciousness is impaired or psychological distress is present. Despite this uncertainty, the dose variable showed a consistent dose-response relationship with mortality across the logistic, Firth, and time-dependent Cox analyses. Fourth, the low incidence of MODS among survivors resulted in a wide confidence interval for its effect estimate in conventional logistic regression. Firth penalized logistic regression provided a more stable estimate and confirmed the robustness of the association, but larger studies are needed to obtain more precise estimates. Fifth, the E-value for the binary NLR contrast was 24.9 for the point estimate and 4.2 for the lower confidence limit, indicating reasonable robustness to unmeasured confounding for this clinically meaningful contrast (VanderWeele and Ding, 2017), although residual confounding cannot be entirely excluded. Sixth, the SHAP analysis was performed on the training set, and the model lacks independent external validation. Seventh, the multivariable model was constructed following LASSO-based variable selection on the same dataset, and the internal bootstrap validation only resampled the final three-variable model rather than the entire analytical pipeline. The reported P values, confidence intervals, and optimism-corrected performance metrics may therefore be optimistically biased. The optimism-corrected AUC of 0.970 should be regarded as likely overestimated, given the limited number of events and the dominant contribution of MODS. The reported calibration intercept and slope were apparent estimates and were not subjected to the same bootstrap-based optimism correction applied to the C-statistic and Brier score. Finally, although MODS was diagnosed within 72 h as an early marker of disease severity, its ascertainment necessarily extends beyond the baseline assessment. A time-dependent Cox regression analysis, in which MODS was treated as a time-varying covariate, confirmed that MODS remained independently associated with mortality after accounting for its time-varying nature, suggesting that prediction leakage did not substantially inflate the model’s performance. Nonetheless, future prospective studies with predefined prediction landmarks are warranted to formally address this concern.

## Conclusion

This single-center retrospective study analyzed the clinical characteristics of patients with intentional diquat self-poisoning. Ingestion dose, MODS, and NLR were strongly associated with increased all-cause mortality. A time-dependent Cox analysis confirmed that MODS remained independently associated with mortality after accounting for its time-varying nature, while an exploratory logistic regression model yielded a clinically applicable prediction equation. When applied after the first 24–72 h of admission, the combined model shows promise as an adjunctive tool for early risk stratification in this single-center cohort. However, given the limited sample size, the exploratory nature of the analysis, and the absence of external validation, these findings should be interpreted with caution. Prospective multicenter studies are warranted before any generalization or consideration of clinical application.

## Data Availability

The raw data supporting the conclusions of this article will be made available by the authors, without undue reservation.
