# Supplementary material for: Combined predictive value of dose, MODS, and NLR for lethal risk of diquat poisoning: a retrospective cohort study
Source: Front Toxicol. 2026 Oct 5;8:1897012. doi: 10.3389/ftox.2026.1897012 (PMC13678667; doi:10.3389/ftox.2026.1897012)
Supplement: Supplementary file 1 [file Supplementaryfile1.docx]

**Supplementary Table S1. Time-dependent Cox regression analysis treating MODS as a time-varying covariate.**

| Variable | HR (95% CI) | P value |
| --- | --- | --- |
| DQ (per 1 mL) | 1.007 (1.002–1.012) | 0.011 |
| NLR (per 1 unit) | 1.015 (0.996–1.033) | 0.119 |
| MODS (time-varying) | 35.24 (13.57–91.50) | < 0.001 |

Schoenfeld residual tests confirmed no violation of the proportional hazards assumption (all *P* > 0.05). The baseline model (DQ + NLR) achieved a time-dependent AUC of 0.932 at 30 days. HR, hazard ratio; CI, confidence interval.

**Supplementary Figure S1. Time-dependent ROC curve at 30 days for the baseline model (Diquat dose + NLR).**
